# Orexin A Suppresses the Growth of Rat C6 Glioma Cells via a Caspase-Dependent Mechanism

**DOI:** 10.1007/s12031-012-9799-0

**Published:** 2012-05-17

**Authors:** Kaja Biegańska, Paulina Sokołowska, Olaf Jöhren, Jolanta B. Zawilska

**Affiliations:** 1 Institute for Medical Biology, Polish Academy of Sciences, 106 Lodowa St., 93-232 Lodz, Poland; 2 Department of Pharmacodynamics, Medical University of Lodz, 1 Muszynskiego St., 90-151 Lodz, Poland; 3 Institute of Experimental and Clinical Pharmacology and Toxicology, University of Lübeck, Ratzeburger Allee 160, 23538 Lübeck, Germany

**Keywords:** Orexin, Hypocretin, Orexin receptors, Cell death, C6 glioma cells

## Abstract

Orexin A and orexin B (also known as hypocretins) are closely related peptides synthesized by hypothalamic neurons. They orchestrate diverse central and peripheral processes by stimulation of two G-protein coupled receptors, OX_1_R and OX_2_R. Recent studies have demonstrated the ability of orexins to promote a robust apoptosis in different cancer cells in culture and a potent growth reduction of human colon tumors in mice xenografts. Here we report effects of orexins on survival of rat C6 glioma cells, an experimental model for studies on glioblastoma multiforme (GBM). Quantitative real-time PCR demonstrated the expression of both types of orexin receptors in C6 cells. Orexin A and orexin B did not affect rat C6 glioma cell proliferation as assessed by [^3^H]thymidine incorporation assay. Incubation of the cells with orexin A (0.001–1 μM) resulted in a marked decrease of cell viability. The observed effect was caspase-dependent, as it was blocked by Z-VAD-fmk, a pan caspase inhibitor. In addition to that, a parallel increase in caspase-3 activity was observed. It is suggested that stimulation of orexin receptors induces death of rat C6 glioma cells through activation of caspase pathway.

## Introduction

Orexins are evolutionarily conserved neuropeptides codiscovered in 1998 by two independent research groups (de Lecea et al. 1998; Sakurai et al. 1998). Orexin A (33 amino acids) and orexin B (28 amino acids) are derived from the cleavage of a common 130-residue (rodent) or 131-residue (human) prohormone, prepro-orexin, and share 46 % amino acid identity in human. Orexin-producing neurons are localized almost exclusively in the dorsal and lateral hypothalamus and project to numerous brain structures. Outside the hypothalamus, orexin-immunoreactive fibers were observed, for example, in the cerebral cortex, olfactory region, thalamus, hippocampus, amygdala, locus coeruleus, raphe nuclei, and area postrema (Nambu et al. 1999). The actions of orexins are mediated by two membrane bound G-protein coupled receptors, OX_1_R and OX_2_R (Sakurai et al. 1998; Kukkonen et al. 2002). It has been recently shown that activated OX_1_R may exist in a homodimeric form (Xu et al. 2011). Orexin A is considered as a high-affinity agonist for OX_1_R, whereas orexin B has significantly lower affinity to OX_1_R. Both peptides show similar affinities to OX_2_R (Sakurai et al. 1998; Ammoun et al. 2003).

Accumulating experimental data indicate that orexins control a number of important physiological processes. They are best known from their stimulatory effect on feeding behavior, regulation of energy homeostasis, sleep–wake cycle, reward-seeking, and drug addiction (Kukkonen et al. 2002; Matsuki and Sakurai 2008; Aston-Jones et al. 2010; Kodadek and Cai 2010). The peptides also control hypothalamic–pituitary–adrenal axis and functions of miscellaneous peripheral organs, including heart, kidney, pancreas, gastrointestinal tract, thyroid, lung, testis, ovaries, and adipose tissue (Voisin et al. 2003; Korczyński et al. 2006; Spinazzi et al. 2006; Heinonen et al. 2008; Okumura and Nozu 2011; Kagerer and Jöhren 2010). The loss or dysfunction of orexin neurons has been shown to cause human and animal narcolepsy (e.g., Chemelli et al. 1999; Lin et al. 1999; Thannickal et al. 2003; Nishino 2007). Recent studies have demonstrated the ability of orexins to induce apoptosis in cancer cells in culture (Rouet-Benzineb et al. 2004; Voisin et al. 2011) and to potently reduce the growth of human colon tumors in mice xenografts (Voisin et al. 2011). It has been proposed that proapoptotic activity is an intrinsic property of orexin receptors (El Firar et al. 2009).

In the present work, we investigated the expression of orexin receptors in rat C6 glioma cells, an experimental model for studies on glioblastoma multiforme (GBM), and analyzed effects of orexins on cell survival. Our results show that orexin A, presumably acting at OX_1_R, suppresses the growth of C6 cells through activation of caspase pathway.

## Materials and Methods

### Rat C6 Glioma Cell Cultures

Rat C6 glioma cell line was obtained from the European Collection of Animal Cell Cultures (Porton Down, UK). The cells were grown in 60-mm Petri dishes in an F-12 Ham Nutrient Mixture supplemented with 10 % FBS Gold serum, 2 mM glutamine, 100 U/ml penicillin, and 100 μg/ml streptomycin in a humidified atmosphere of 95 % air and 5 % CO_2_ at 37 °C. For subcultures, cells were harvested in trypsin–EDTA solution twice a week and seeded at a density of 10^6^ cells per dish. For experimentation, the cells between passages eight and 20 were used.

### Real-Time Quantitative RT-PCR

Total RNA was extracted from C6 glioma cells by using TRI Pure Isolation Reagent (Roche, Meylan, France) according to the manufacturer's instruction. For each sample, total RNA (1 μg) was subjected to reverse transcription (RevertAid H Minus First Strand cDNA Synthesis Kit; Fermentas, Burlington, Canada) according to the manufacturer's specifications. Specific sense and antisense oligonucleotide primers for amplification of mRNAs of rat OX_1_R and OX_2_R were obtained from Invitrogen (Karlsruhe, Germany). The sequences of specific primers and the procedure of real-time quantitative PCR (qPCR) were published previously (Jöhren et al. 2001). Shortly, 2 μl of the first strand cDNA reaction was incubated in the presence of 3 mM MgCl_2_; 200 μM of dGTP, dATP, dCTP, and dUTP; Platinum Taq DNA Polymerase; fluorescence dye SYBR green I; and the appropriate sense and antisense primers in a final volume of 25 μl (Platinum® SYBR® Green qPCR SuperMix, Invitrogen) on the 7000 Sequence Detection System of Applied Biosystems (Darmstadt, Germany). Each sample was analyzed in duplicate along with standards and no template controls. Product purity was regularly confirmed for each sample by dissociation curve analysis. Copy number calculations were based on the cycle threshold method (Higuchi et al. 1993). Serial dilutions of known amounts of specific cDNA fragments were used to generate standard curves. The threshold cycle number (CT) for each sample was calculated using the 7000 Sequence Detection System software with an automatic baseline setting and a fluorescence threshold (Rn) of 0.2.

### Cell Viability Assay

C6 glioma cells (seeded at a density of 30,000 cells/well) were grown in 96-well plates in standard culture conditions. Twenty-four hours before exposing cells to the tested chemicals, the culture medium was replaced with fresh serum-free medium. Cell viability and mitochondrial function were measured by 3-(4,5-dimethyl-2-thiazolyl)-2,5-diphenyl-2*H*-tetrazolium bromide (MTT) reduction to MTT formazan by cellular mitochondrial dehydrogenases. Following exposure to orexin A and orexin B for 24 and 48 h, the cell cultures were washed in PBS before the addition of MTT (0.5 mg/ml) and incubated for 3 h at 37 °C. Formazan crystals were solubilized in dimethyl sulfoxide (DMSO; 100 %) and absorbance, proportional to the number of viable cells, was measured at 570 nm using a microplate reader (EnVision 2103, PerkinElmer).

### [*Methyl*-^3^H]Thymidine Incorporation Assay

Rat C6 glioma cells were seeded on 96-well plates (30,000 cells/well) and cultured in the standard conditions for 24 h to allow cell adhesion. The medium was then removed; cells were washed with PBS, and cultured for another 24 h with serum-free medium. Thereafter, orexin A and orexin B were added in serum-free medium, and cells were cultured for 24 or 48 h in the presence 0.5 μCi of [*methyl*-^3^H]thymidine during the last 18 h of incubation. The medium was then removed, and the cells were washed twice with PBS, harvested, and solubilized in 0.1 % Tween 20. The radioactivity of [*methyl*-^3^H]thymidine incorporated into DNA of dividing cells was counted by a β-plate liquid scintillation counter (MicroBeta TriLux, Luminescence Counter, PerkinElmer).

### In Situ Caspase Activation

Rat C6 glioma cells were seeded at a density of 10^6^ on poly-L-ornithine (0.01 mg/ml) coated 60-mm Petri dishes and grown for 24 h in standard culture conditions. The culture medium was then replaced with fresh serum-free medium, and the cells were cultured for another 24 h. Thereafter, the medium was replaced with fresh serum-free medium, containing or not orexins. After 24 h, caspase activation was detected using the EnzChek Caspase-3 Assay Kit from Invitrogen Life Science (Warsaw, Poland) according to the manufacturer's information.

### Chemicals

Nutrient Mixture F-12 Ham medium, poly-L-ornithine, DNase I, trypsin, glutamine, penicillin, streptomycin, and MTT were from Sigma-Aldrich (Poznan, Poland). FBS Gold was from PAA Laboratories (Cölbe, Germany). dGTP, dATP, dCTP, and dUTP, and Platinum Taq DNA Polymerase were from Invitrogen (Karlsruhe, Germany). Orexin A and orexin B were from PolyPeptide Laboratories (Strasbourg, France). Z-VAD-fmk (benzyloxy-carbonyl-Val-Ala-Asp(OMe)-fluoromethylketone) was purchased from Tocris Bioscience (Bristol, UK). Petri dishes and multiwell plates for cell cultures were from Nunc (Wiesbaden, Germany). [*Methyl*-^3^H]thymidine (specific activity 6.7 Ci/mmol) was from PerkinElmer Life Sciences (Boston, MA, USA). Other chemicals were of analytical purity and were obtained mainly from Sigma-Aldrich (Poznań, Poland).

### Data Analysis

Data are expressed as mean ± standard error of the mean (SEM) values and were analyzed for statistical significance by one-way ANOVA followed by post hoc Student–Newman–Keul's test using Prism version 5 (GraphPad, San Diego, CA, USA).

## Results

### Expression of Orexin Receptors in Rat Glioma C6 Cell Line

**Fig. 1:**
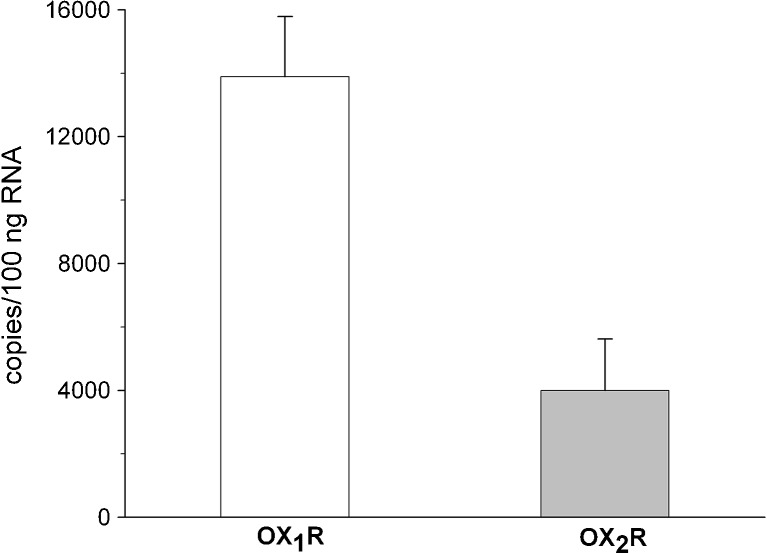
Expression of OX_1_R and OX_2_R mRNA in the rat C6 glioma cell line. Results are expressed as specific mRNA copy numbers per 100 ng of total RNA and represent means ± SEM of eight values per group

Quantitative RT-PCR analysis with specific primers revealed the presence of both types of orexin receptors in rat C6 glioma cell line (Fig. 1). The specificity of quantitative RT-PCR was confirmed by subsequent agarose gel analysis, showing bands specific for OX_1_R, OX_2_R, and GAPDH (data not shown).

### Effects of Orexins on Viability of Rat C6 Glioma Cells

**Fig. 2:**
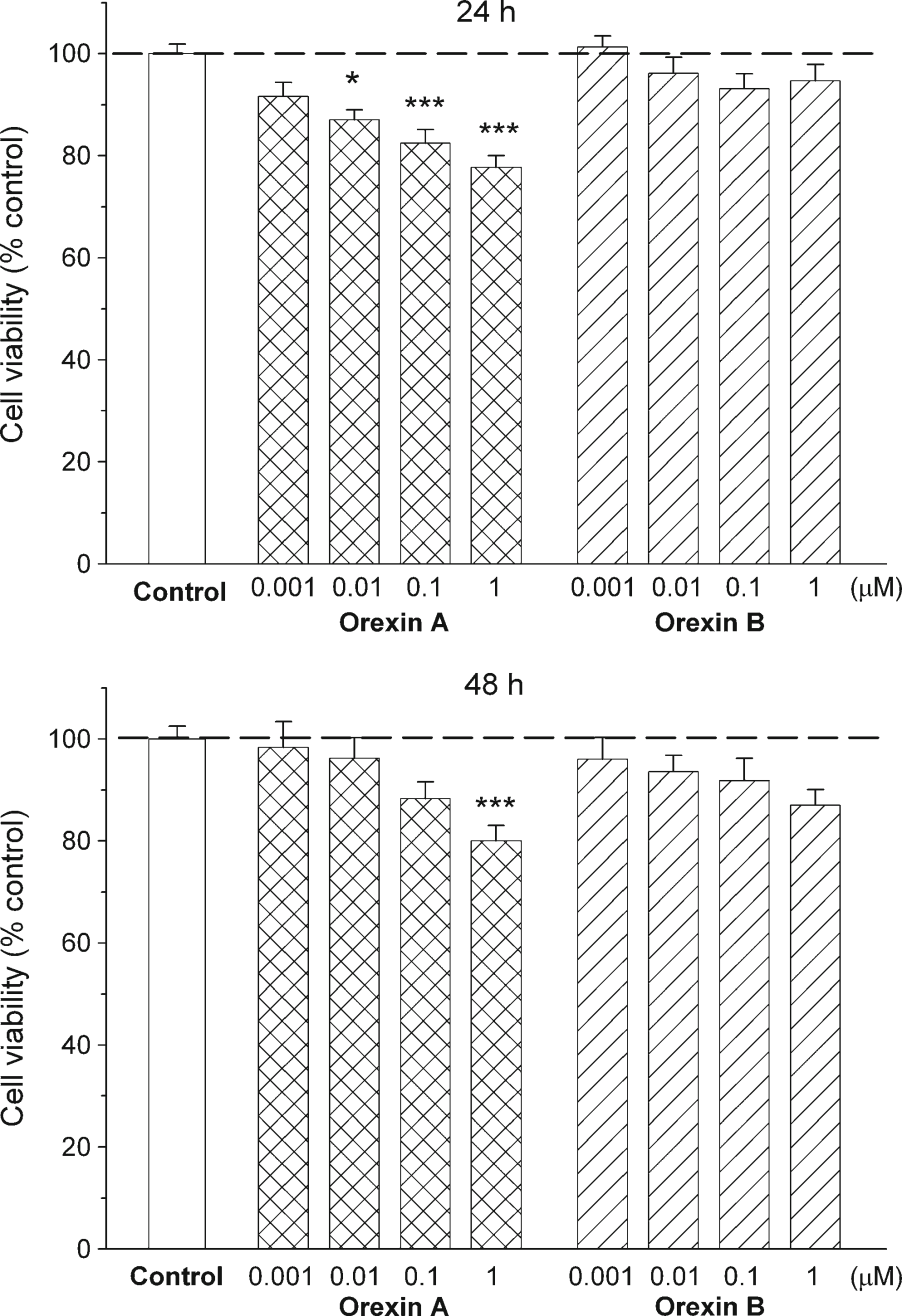
Effects of orexin A and orexin B on viability of rat C6 glioma cells. The cells were incubated with peptides (0.001–1 μM) for 24 h (*top*) or 48 h (*bottom*). The viability of cells was assessed with the aid of MTT test. The results are expressed as a percent of the respective control values and represent means ± SEM of nine to 59 values per group. *Asterisk* indicates *P* < 0.05, *two asterisks* indicate *P* < 0.01, *three asterisks* indicate *P* < 0.001 versus control

To determine effects of orexins on cell survival, C6 glioma cells were stimulated with various concentrations (0.001–1 μM) of orexin A and orexin B and examined by MTT assay. To avoid complex effects from growth factors and other mediators present in serum, the cells were serum-starved for 24 h prior to exposure to the tested compounds. Rat C6 glioma cells are viable in serum-free medium, and the mean values of absorbance units (A_572_) were only slightly lower compared to cells cultured in standard conditions (0.35 ± 0.02 versus 0.38 ± 0.01, *n* = 10–12/group, for cells cultured without serum and cells cultured with serum, respectively). Incubation of rat C6 glioma cells with 0.001–1 μM of orexin A for 24 h decreased the number of viable cells. The observed effect was concentration-dependent, with 1 μM of orexin A being the most potent (decrease by 22 % below the control). The antisurvival effect of orexin A was statistically significant in concentrations ranging from 0.01 to μM (Fig. 2 top). The calculated IC_50_ value for orexin A was 4.7 nM. When the exposure time had been prolonged from 24 to 48 h, a statistically significant decrease (by 20 %) in cell viability was seen only at the highest concentration of orexin A used, i.e., 1 μM (Fig. 2 bottom). Orexin B showed a tendency to decrease cell survival, after both 24 and 48 h, but the level of inhibition did not reach statistical significance (Fig. 2).

### Orexins Did Not Affect Proliferation of Rat C6 Glioma Cells

**Fig. 3:**
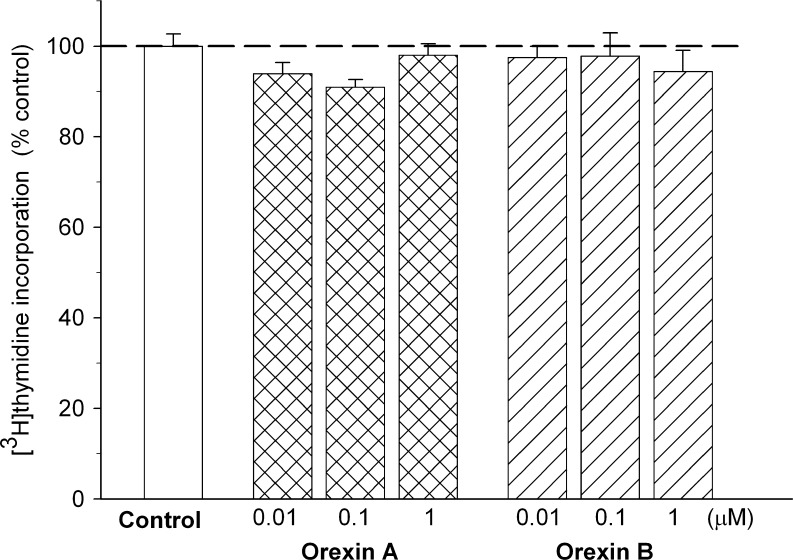
Effects of orexin A and orexin B on proliferation of rat C6 glioma cells. The cells were incubated with peptides (0.001–1 μM) for 24 h. Proliferative activity of the cells was assessed by [*methyl*-^3^H]thymidine incorporation assay. The results are expressed as a percent of the mean control value (3,463 cpm) and represent means ± SEM of five to six values per group

To determine whether the orexin-induced decrease in cell survival was due to the inhibition of rat C6 glioma cells proliferation, we evaluated effects of the peptides on [*methyl*-^3^H]thymidine incorporation into the cells. Both orexin A and orexin B were ineffective in reduction of the cell proliferation rate (Fig. 3).

### Orexin A-Induced Death of Rat C6 Glioma Cells Required Activation of Caspase Pathway

**Fig. 4:**
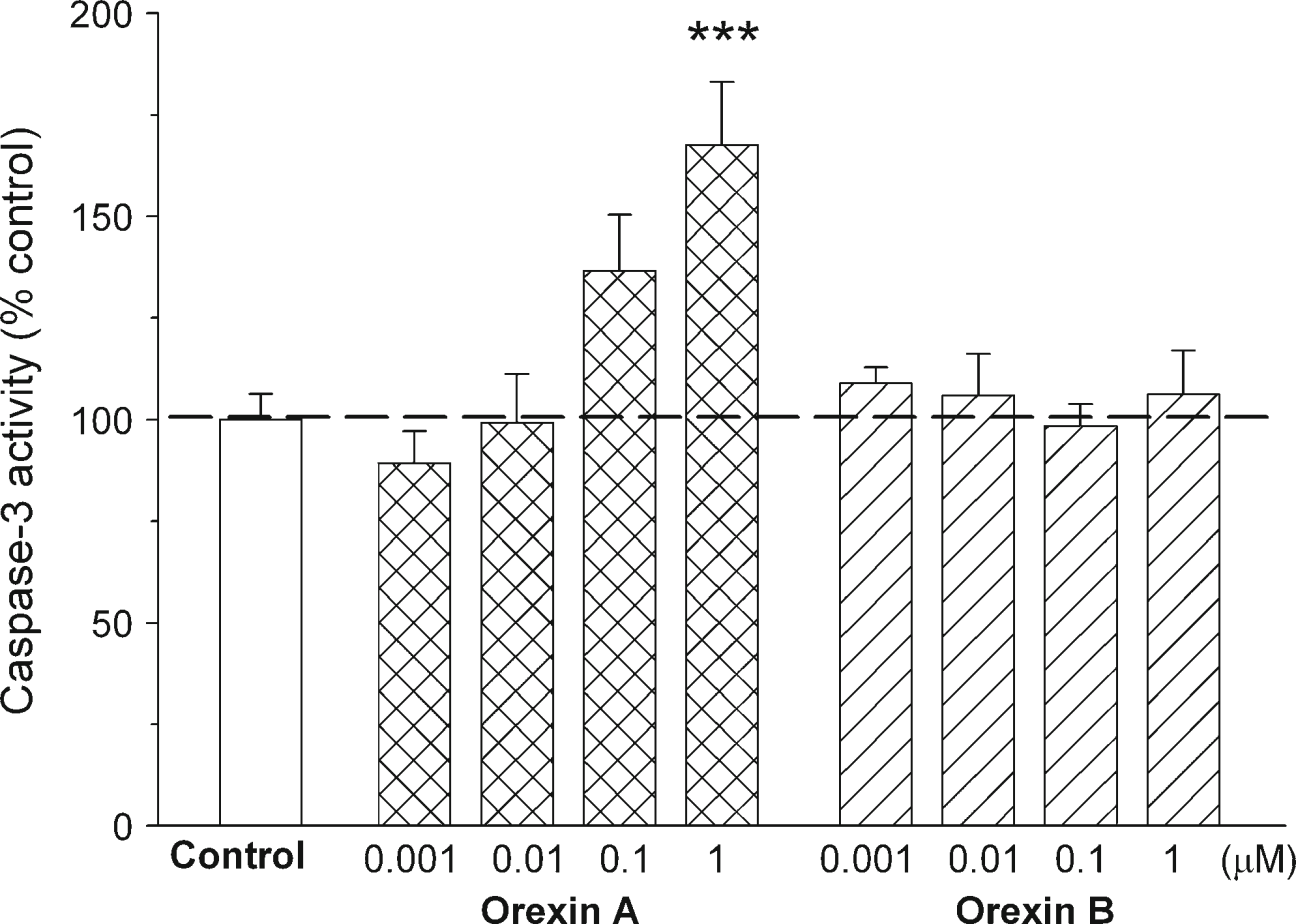
Effects of orexin A and orexin B on basal caspase-3 activity in rat C6 glioma cells. The cells were incubated with the peptides (0.001–1 μM) for 24 h. The results are expressed as a percent of the respective control values and represent means ± SEM of five to 13 values per group. *Three asterisks* indicate *P* < 0.001 versus control

**Fig. 5:**
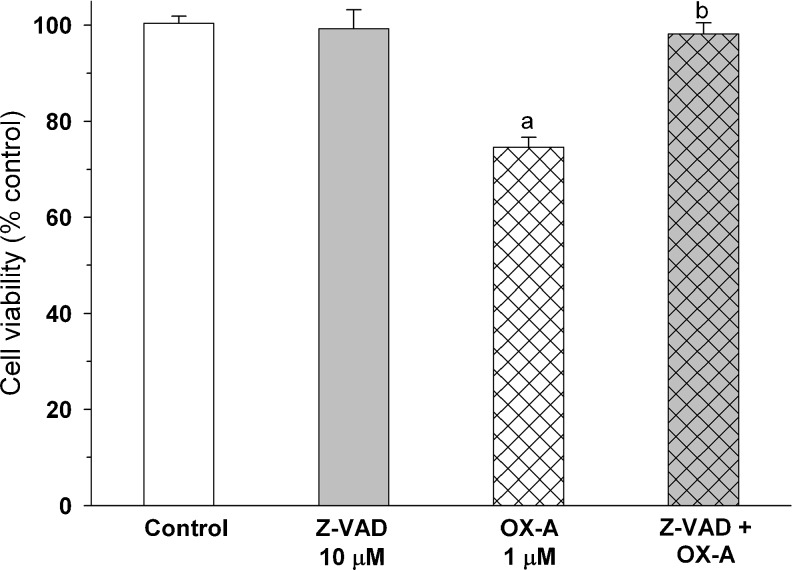
Effects of Z-VAD-fmk (*Z-VAD*), a pan caspase inhibitor, on decrease in viability of rat C6 glioma cells induced by 24-h exposure to orexin A (*OX-A*). The results are expressed as percent of control values and represent means ± SEM of 15–43 values per group. *Letter a* indicates *P* < 0.001 versus control, *letter b* indicates *P* < 0.001 versus orexin A

To test whether activation of caspase pathway is involved in the orexin A-evoked death of rat C6 glioma cells, caspase-3 activity was measured. The obtained results revealed concentration-dependent activation of the enzyme (Fig. 4). The effect of 1 μM orexin A reached the level of statistical significance, increasing the caspase-3 activity by 67 % above control. To confirm the importance of caspase-dependent processes in the studied action of orexin A, a set of experiments with Z-VAD-fmk, a pan caspase inhibitor, were conducted. This compound, used at a 10 μM concentration, did not affect the survival of C6 cells. When Z-VAD-fmk was added to the incubation medium prior to orexin A (1 μM), it fully blocked the suppressive effect of the peptide on viability of rat C6 glioma cells as measured by MTT assay (Fig. 5).

## Discussion

The present work demonstrates, for the first time, that orexin receptors, OX_1_ and OX_2_, are expressed at the mRNA level in the rat C6 glioma cell line. In a search for a potential role of orexins in C6 glioma cells, we examined effects of the peptides on their survival. Orexin A suppressed the growth of the tested cell line. On the contrary, orexin B did not significantly alter cell viability. Orexin A is considered as a high-affinity agonist for OX_1_R, whereas orexin B has a significantly lower affinity to OX_1_R. Both peptides show similar affinities to OX_2_R (Sakurai et al. 1998; Ammoun et al. 2003). Thus, it can be hypothesized that activation of OX_1_R induces death of rat C6 glioma cells; however, the involvement of OX_2_R in the studied action of orexin A cannot be excluded.

The negligible effect of orexin B (in concentrations up to 1 μM) on survival of C6 glioma cells might reflect ligand profiling of orexin receptors in these cells, as originally postulated by Putula et al. (2011). In studies on heterologous cell lines expressing human orexin receptors, they demonstrated that the potency of orexinergic agonists (orexin A, orexin B, and [Ala^11^,*D*-Leu^15^]orexin B) is related not only to the cell type (receptor expression level, coupling efficiency, and signal transduction pathways) but also to agonist trafficking of receptor responses (so-called signal trafficking). In accordance with our hypothesis are data demonstrating that in human adrenal cortical cells (expressing almost solely OX_1_ receptors), orexin A stimulated cortisol secretion with an EC_50_ value around 0.2 nM, while orexin B was ineffective at concentrations up to 1 μM (Mazzocchi et al. 2001). Orexin A, but not orexin B (also used at 1 μM), concentration dependently increased corticosterone secretion and enhanced cyclic AMP production from rat adrenocortical cells (Ziolkowska et al. 2005).

Studies on mechanisms by which orexin A reduced C6 cell survival revealed that the decrease in the number of cells was not associated with an inhibition of their proliferation rate, as the peptide did not alter this process. Two lines of evidence suggest that rat C6 glioma cells' death is associated with activation of caspase pathway: (1) orexin A increased basal caspase-3 activity and (2) incubation of cells with pan caspase inhibitor suppressed the orexin A-induced inhibition of cell survival. The antisurvival action of orexins has been previously demonstrated in experiments conducted on CHO cells transfected with human OX_1_R (Ammoun et al. 2006) and OX_2_R cDNA (Voisin et al. 2006). Further studies performed on cell lines derived from human brain (SK-N-MC) and colon (HT29-D4, Caco-2, SW480, and LoVo) tumors revealed that the cells die through apoptosis induction. Notably, although orexins induced apoptosis in colon cancer cells, the peptides were unable to trigger this process in normal colonic epithelial cells which, in contrast to the neoplastic cells, do not express native orexin receptors (Rouet-Benzineb et al. 2004). Elegant studies performed by the group of M. Laburthe revealed that the ability to induce apoptosis is an intrinsic property of orexin receptors (Rouet-Benzineb et al. 2004; Voisin et al. 2006; Laburthe et al. 2010). They identified two tyrosine-based motifs within the structure of OX_1_R, i.e., immunoreceptor tyrosine-based motif (ITIM) and immunoreceptor tyrosine-based switch motif (ITSM), and demonstrated that phosphorylation of tyrosines within these motifs, followed by a recruitment of the tyrosine phosphatase SHP-2 and the subsequent activation the enzyme, are essential steps required for apoptosis driven by orexins. Downstream events include release of cytochrome c from mitochondria and activation of caspase-3 and caspase-7 (El Firar et al 2009; Laburthe et al. 2010).

Although the expression level of orexin receptors in C6 glioma cells was similar to that found in astrocytes from rat cerebral cortex, orexins had negligible effects on survival of the latter cell type (Biegańska K, unpublished observation). Interestingly, orexin A and orexin B, with comparable potencies, suppressed caspase-3 activity and increased viability of cultured rat cortical neurons (Sokołowska et al. 2012). In these cells, the expression level of OX_2_R is significantly higher than those observed in rat astrocytes and C6 glioma cells, a phenomenon that may determine different effects of orexin B on cell viability in the rat brain (Urbańska et al. 2012). A question arises as to molecular mechanisms underlying dual functions of orexins in the context of cell growth and survival. Why do the peptides exert antisurvival activity in one cell type and prosurvival activity in another? One likely explanation to this might be an intrinsic sensitivity of cells to the action of cytochrome c. In support of this hypothesis are data demonstrating that primary neurons from the mouse cerebellum and cerebral cortex are remarkably resistant to cytosolic cytochrome c, whereas tumor tissue from mouse models of both high-grade astrocytoma and medulloblastoma display hypersensitivity to cytochrome c when compared with the surrounding brain tissue. Furthermore, it has been shown that cytosolic cytochrome c is sufficient to induce apoptosis in various human neoplastic cell lines: neuroblastoma (SH-SY5Y), glioblastoma (MGR3, MGR1, D54MG, D247MG, and H392), and medulloblastoma (UW228, D341MED, and MCD1). This differential sensitivity to cytochrome c is attributed to high Apaf-1 levels in the tumor tissue compared with low levels in the adjacent brain tissue (Johnson et al. 2007). In the intrinsic pathway of apoptosis, cytochrome c released from mitochondria, binds to Apaf-1, leading to recruitment of procaspase-9 and formation of the apoptosome. Apoptosome-mediated activation of caspase-9 activates caspase-3 and caspase-7, which promote cell death (Danial and Korsmeyer 2004). Another possible factor determining the impact of orexins on cell viability is activation of mitogen-activated protein kinase (MAPK) signaling pathways. Studies performed on human embryonic kidney (HEK-293) and CHO cells stably expressing human OX_1_R and OX_2_R have demonstrated that orexins can exert opposite effects on cell survival through activation of the classical MAPK pathways. The ERK1/2 pathway was considered to be protective, whereas p38 was central for cell death (Ammoun et al. 2006; Tang et al. 2008). In accordance with these observation are results of our preliminary experiments demonstrating that the suppressive action of orexin A on survival of rat C6 glioma cells was blocked by SB 202190, a specific p38 MAPK inhibitor, whereas the orexins-induced increase in viability of cortical neurons was abolished by U0126, a MEK inhibitor (Biegańska K, Sokołowska P and Zawilska JB, unpublished data).

Our work was done on rat C6 glioma cell line, a widely used experimental model for studies on GBM. This cell line is derived from N,N′-nitromethylurea-induced tumors in random-bred Wistar–Furth rats (Benda et al. 1968). When the rat glioma C6 cells are injected into the brain of newborn rats, they form tumors morphologically similar to GBM (Auer et al. 1981). GBM is the most common and aggressive type of malignancy in the central nervous system, and despite the latest progress in pharmacological and radioactive therapeutic approaches, it is still resistant to these treatments and generally considered as an incurable disease (Lim et al. 2011; Cimini and Ippoliti 2011). A median survival time remains approximately 15 months for newly diagnosed patients and 7 months for recurrent/relapsed GBM (Henriksson et al. 2011). Taking into account the above facts, searching for compounds capable of inducing glioma cells' death appears of extreme importance. Along this line, the antisurvival action of orexin A described in this work, together with the data demonstrating the ability of orexins to promote a robust apoptosis in different cancer cells in culture and a potent growth reduction of human colon tumors in mice xenografts (Rouet-Benzineb et al 2004; Voisin et al. 2011) points to orexin receptors as new and promising targets in studies on anticancer therapies.

In conclusion, we provided the first evidence for the presence of orexin receptors in rat C6 glioma cells. Furthermore, we demonstrated that activation of orexin receptors by orexin A induces caspase-dependent cell death. The molecular mechanism(s) by which orexin receptors mediate C6 cell death, including the intracellular signaling pathways, remains to be elucidated.

## Abbreviations

GBM: Glioblastoma multiforme
MAPK: Mitogen-activated protein kinase
MEK: MAPK/ERK kinase
MTT: 3-(4,5-Dimethyl-2-thiazolyl)-2,5-diphenyl-2*H*-tetrazolium bromide
OX_1_R: Type 1 orexin receptor
OX_2_R: Type 2 orexin receptor
SB 202190: 4-[4-(4-Fluorophenyl)-5-(4-pyridinyl)-1*H*-imidazol-2-yl]phenol
U0126: 1,4-Diamino-2,3-dicyano-1,4-bis[2-aminophenylthio]butadiene
Z-VAD-fmk: Benzyloxy-carbonyl-Val-Ala-Asp(OMe)-fluoromethylketone
